# Adaptation of the Short Version of Santa Clara Strength of Religious Faith Questionnaire in Türkiye

**DOI:** 10.1007/s10943-025-02262-1

**Published:** 2025-01-30

**Authors:** Gülcan Bahcecioglu Turan, Zülfünaz Özer, Nisa Yavuzer Bayrak

**Affiliations:** 1 Department of Nursing, Faculty of Health Sciences, Fırat University, Elazığ, Turkey; 2 Department of Nursing, Faculty of Health Sciences, Istanbul Sabahattin Zaim University, Istanbul, Turkey; 3 Department of Nursing, Faculty of Health Sciences, Nevsehir Haci Bektas Veli University, Nevşehir, Turkey

**Keywords:** Santra Clara, Religion, Faith, Spirituality, Nursing

## Abstract

The present study was conducted to adapt the short version of the Santa Clara Strength of Religious Faith Questionnaire into Turkish and to examine the validity and reliability of the scale. This methodological study was conducted between January and May 2024 with 283 individuals aged 18 and older in Turkey. Data were collected online by using “Personal Information Form” and the “Short Version of the Santa Clara Strength of Religious Faith Questionnaire”. It was found that all participants believed in God. According to the study results, item-based content validity index (I-CVI) was between 0.87 and 1.00, and the scale-based content validity index (S-CVI) was found to be 0.98. Factor loadings of the scale items vary between 0.570 and 0.840. Fit indices of the scale were calculated as follows: *X*^2^ = 9.64, df = 4 (p < 0.05), *X*^2^/df = 2.41, RMSEA = 0.071, CFI = 0.99, SRMR = 0.021, TLI = 0.98, RMR = 0.012, AIC = 31.64. The overall Cronbach’s alpha value of the scale was 0.860, and the Omega reliability value was 0.866. Short Version of the 5-item Santa Clara Strength of Religious Faith Questionnaire was validated without any changes to the original form. The Turkish version of the Short Version of the Santa Clara Strength of Religious Faith Questionnaire is a valid and reliable instrument for measuring individuals’ strength of religious faith.

## Introduction

Religious faith is the factors that shape the values, worldviews, and lives of individuals and constitute the cultural characteristics of society. It is the guide that helps people make sense of their lives, guide their decisions, and shape their relationships with other people. To begin with, religious faith plays an important role in the formation of personal identity and the development of a sense of life purpose. For many people, faith serves as the basis for values and ethics, influencing behaviour and decision-making (Caycho-Rodríguez et al., 2022; Jackson et al., 2021; Susanto et al., 2023).

Religious faith shapes not only the behaviour of individuals, but also family relationships and social cohesion. Respect for faith and traditions is often associated with stronger family ties and strong social support (Achour et al., 2021; İlerisoy, 2022; Regnerus & Burdette, 2006). Religious faith plays an important role in determining and enforcing social norms and rules. This helps to build resilience in coping with challenges in parenting practices by strengthening social support networks, especially in times of personal and societal crisis (El-Khani et al., 2023; İlerisoy, 2022).

In addition to establishing rules and norms in society, religious faith also plays an important role in the process of identity development and adding meaning to individuals’ lives. Research shows that religious individuals are able to find meaning and purpose in life and this shapes their actions and social interactions (Jorgensen et al., 2016; Storm, 2016). This also has a similar effect on professional behavioural patterns, and it is emphasised that cultural and religious faith of healthcare professionals can significantly affect their clinical practices and decisions (Al-Yateem et al., 2022).

In recent years, the important effects of religious beliefs on health have gained more and more attention. These beliefs, which have been discovered to have positive effects on both physical and mental health, also manifest themselves in a different field (Koenig & Carey, 2024). Research shows that religious beliefs have the potential to improve health outcomes (Oh et al., 2022; Öztürk et al., 2024; Roth et al., 2016; Turan et al., 2023). It is emphasised that religious belief has positive effects on the recovery process and quality of life in patients with acute myocardial infarction. Similarly, religious faith has been reported to make a positive contribution to social health and to have beneficial effects on general mental health (Blanchard et al., 2008; Kim et al., 2009; Roth et al., 2016).

Religious faith is reported to have a strong relationship with favourable health outcomes and this is of great importance for nursing practice. The impact of religious faith should be evaluated in improving patient care, addressing the individual in all aspects and providing guidance on ethical dilemmas faced in clinical settings (Erami & Taghadosi, 2023; Martins et al., 2024; Tanriverdi, 2021). On the other hand, studies have shown that religious beliefs of nurses can be effective in the processes of making and implementing care decisions (Davis et al., 2012; Toro-Flores et al., 2019; Wu et al., 2020). In addition, nurses should be aware of the power of patients’ religious beliefs and be sensitive to this in order to provide more effective care (Hickerson et al., 2019; Martins et al., 2024).

The multidimensional nature of religious faith emphasises its important role in human life, leading to the need for further research and in-depth understanding at both academic and practical levels. For this reason, it has been stated that religious faith has multidimensional effects on psychological, emotional, and social situations and various measurement tools have been developed to measure this effect. The Santa Clara Strength of Religious Faith Questionnaire (SCSRFQ) is a basic tool that aims to measure the strength of religious faith among different populations and belief systems (Caycho-Rodríguez et al., 2022; Plante, 2010).

The Santa Clara Strength of Religious Faith Questionnaire (SCSRFQ) is a tool that provides a comprehensive assessment of the strength of religious faith, both in its original ten-item version and in its five-item short form. The availability of a large number of forms in different cultures and languages in the fields of health, education, and psychology facilitates its use (Akin et al., 2015; Cummings et al., 2015; Dianni et al., 2014; Lewis, 2021; Lucchetti et al., 2013; Pakpour et al., 2014; Plante et al., 2002). The Santa Clara Strength of Religious Faith Questionnaire Short Version (SCSRFQ) is an ideal tool in terms of ease of use and suggests that religious faith in patients and healthy individuals can play a guiding role both at the individual level and in holistic nursing care. It is important to evaluate the strength of religious faith in sick and healthy individuals. In this context, the translation of the short version of the Santa Clara Strength of Religious Faith Questionnaire into Turkish will allow us to better understand the impact of individuals’ religious beliefs on their lives and the decisions they make.

### Study Questions

- Is the “Short version of the Santa Clara Strength of Religious Belief Survey” a valid scale for Turkish society?
- Is the “Short version of the Santa Clara Strength of Religious Belief Survey” a reliable scale for Turkish society?

## Methods

### Design

The study is a methodological research.

### Participants and Sample

Data collection forms prepared with GoogleDocs program were sent online (e-mail, social media) to individuals over the age of 18 in Turkey between January and May 2024, and they were asked to fill out the forms and share them with individuals aged 18 and older around them. A total of 643 people were reached with the online survey form. Since 250 people did not meet the inclusion criteria (being 18 years of age, belief in a religion, or older and completing the survey form in 1–2 min) and 110 people did not consent to answering the survey, the study was completed with 283 people. It has been stated that the sample size in scale adaptation studies should be at least 5 times (10 times if possible) the number of scale items (DeVellis & Thorpe, 2021; Seçer, 2020). The original form of the BV-SCSRFQ consists of five items. Therefore, the sample size was presumed to be at least 25 or 50. Therefore, the study was finalised with 283 individuals who accepted to participate in the study and met the research criteria.

### Data Collection Tools

Data collection tools of the study consisted of “Personal Information Form” and “The Brief Version of the Santa Clara Strength of Religious Faith Questionnaire”.

### Personal Information Form

This form consists of questions on age, gender, educational status, marital status, employment status, presence of chronic disease, and disease diagnosis in those with chronic disease.

### The Brief Version of the Santa Clara Strength of Religious Faith Questionnaire

It was developed by Plante and Boccaccini (1997) with 10 items to determine the level of strength of religious faith in individuals (Plante & Boccaccini, 1997). In 2002, a 5-item short version was developed again by Plante et al. The scale is a 4-point (strongly disagree = 1, strongly agree = 4). Likert-type and unidimensional scale consist of five items. There are no reverse coded items in the scale. The scores taken from all items of the scale are summed to obtain the individual’s strength of religious faith score. The range of possible scores that can be obtained from the scale varies between 5 and 20. High scores from the scale indicate that the strength of religious faith is high (Plante et al., 2002).

### Adaptation Process

Adaptation of the scale was carried out in three stages.

#### First Stage

The scale form was translated into Turkish by two English language experts. The scale form was sent to five experts (one public health nursing specialist, two nursing principles specialist, two internal medicine nursing specialist, one theology graduate and specialist, and one Turkish language expert). CVI was calculated and since there were no items with a score below 0.80, no item was omitted. After the expert opinions, some items were revised and translated back into English by two foreign language experts (Koenig & Zaben, 2021). The initial and final versions of the form were compared and it was determined that there was no significant difference. The pilot application phase was started.

#### Second Stage (Pilot Application)

In the pilot application stage, the item correlation value of the scale items should be > 0.30 and Cronbach’s alpha value should be > 0.70 (DeVellis & Thorpe, 2021; Seçer, 2020; Shrestha, 2021). Thirty individuals were reached in the pilot application phase. It was determined that the item correlation value of all items was > 0.30. In addition, since the Cronbach’s Alpha value of the scale was > 0.70, no item was omitted in the pilot application phase and the main application phase was started with five items.

#### Third Stage (Main Implementation)

For data collection, items were uploaded to ‘Google forms’ and a link was created. The link was distributed as an online form (e-mail, social media). The study was carried out with 283 individuals who were reached by online form on the specified dates and who accepted to participate in the study. Confirmatory factor analysis and reliability analyses of the scale were conducted. The fit indices obtained in the confirmatory factor analysis were analysed in line with the literature (Bae, 2017; Woo, 2017). The values found showed that the scale structure was valid. It is recommended in the literature to conduct the retest after 15–30 days (Alpar, 2022; Seçer, 2020; Koenig & Zaben, 2021). In the study, the retest was conducted 15 days later.

### Statistical Analysis

The data were transferred from ‘Google Forms’ to EXCEL file. At this stage, the data were arranged to be transferred to the SPSS software. The data were analysed with SPSS 27 and LISREL software. Item total correlation values and total Cronbach’s alpha values of the scale items were analysed. The suitability of the dataset and sample size for analysis was determined by Kaiser Meyer Olkin (KMO) and Barlet’s sphericity test. A KMO value > 0.60 and a significant Barlet’s test of sphericity indicate the adequacy of the sample and the suitability of the data set for analysis (Seçer, 2020). In order to ensure the construct validity of the scale, the data were transferred to the Lisrel software package and fit indices (Relative Chi-square index (CMIN/DF), root mean square error of approximation (RMSEA), comparative fit index (CFI), Tucker Lewis index (TLI) standardised root mean square residual (SRMR), and root mean square residual (RMR) values were examined. A CMIN/DF value of < 5, a RMSEA value of < 0.08, a CFI value of > 0.90, a SRMR value and RMR value of < 0.08, and a TLI value of > 0.90 indicate that the fit indices of the scale are acceptable (Bae, 2017; Byrne, 2013; Seçer, 2020; Woo, 2017) Cronbach’s alpha coefficient and McDonald Omega reliability analysis results were analysed to determine the reliability of the scale. A reliability coefficient > 0.70 indicates that the scale is reliable (Pallant, 2020; Seçer, 2020). In addition, retest method was used to determine the invariance of the scale against time (Seçer, 2020; Koenig & Zaben, 2021).

### Ethical Considerations

Approval was received from the Ethics Committee of a university and institutional permission (2023/ 13–30 numbered) was obtained from the hospital where the study would be conducted. Official permission was taken from the owner of the scale via e-mail to adapt the ISDS used in the study into Turkish. The study was conducted in accordance with the principles of the Helsinki Declaration of Human Rights. Verbal and written consent was obtained from the individuals who participated in the study after explaining the purpose of the study.

## Results

The age range of the participants was 18–85 years, and the mean age was 42.38 ± 17.12 years. It was found that 58.7% of the participants were female, 54.1% were ill, 32.2% were primary school graduates, 48.4% were not working, 55.5% had an income equal to their expenses, 55.9% had a chronic disease, and 7.1% of those with chronic diseases had diabetes. It was also found that the religious belief of all participants was belief in God (Müslim) (Table 1).

**Table 1:** Descriptive characteristics of the participants (n = 283)

Characteristics	Number (*n* = 283)	%
Gender		
Female	166	58.7
Male	117	41.3
Marital status		
Married	153	54.1
Single	130	45.9
Educational status		
Literate	9	3.2
Primary education	91	32.2
Secondary Education	56	19.8
High School	50	17.7
Undergraduate and above	77	27.2
Employment status		
Yes	145	51.6
No	138	48.4
Income status		
Income < expense	59	24.4
Income = expense	157	55.5
Income > expense	57	20.1
Presence of chronic disease		
Yes	127	44.9
No	156	55.1
Name of the chronic disease (127)		
Asthma	18	6.4
COPD	19	6.5
Hypertension	25	5.3
Diabetes	30	7.1
Heart failure	15	5.3
Cancer	20	7.1
Religious faith		
Müslim	283	100
	Mean ± SD	Min–Max
Age (yr)	42.38 ± 17.12	18–85

### Results Related to Validity

#### Content Validity

In the study, the item-based content validity index (I-CVI) was between 0.87 and 1.00, while the scale-based content validity index (S-CVI) was 0.98.

#### Construct Validity

Before the construct validity, KMO and Barlett’s Sphericity Test were conducted to check the suitability of the sample size and the suitability of the dataset for analysis. KMO value was found to be 0.850. Barlett’s Sphericity Test was found to be significant (*x*^2^ = 682.227; *p* = 0.000). In order to show the validity of the existing 5-item unidimensional structure, CFA analysis was conducted before EFA. As a result of the CFA analysis, it was found that the factor loadings ranged between 0.570 and 0.840 and the unidimensional structure was confirmed (Table 2).

**Table 2:** Mean, item correlation coefficient, and CFA factor load results

Scale items		Mean ± SD	Corrected item total correlations	Factor loadings
				*F*1
Item 1	I pray daily	3.07. ± 0.85	0.578	0.570
Item 2	I look to my faith as providing meaning and purpose in my life	3.25 ± 0.75	0.755	0.780
Item 3	I consider myself active in my faith or church	2.84 ± 0.77	0.671	0.740
Item 4	I enjoy being around others who share my faith	3.19 ± 0.70	0.727	0.840
Item 5	5. My faith impacts many of my decisions	3.13 ± 0.73	0.743	0.830
Cronbach’s alpha: 0.860				
Omega reliability values: 0.866				

### Confirmatory Factor Analysis

**Fig. 1:**
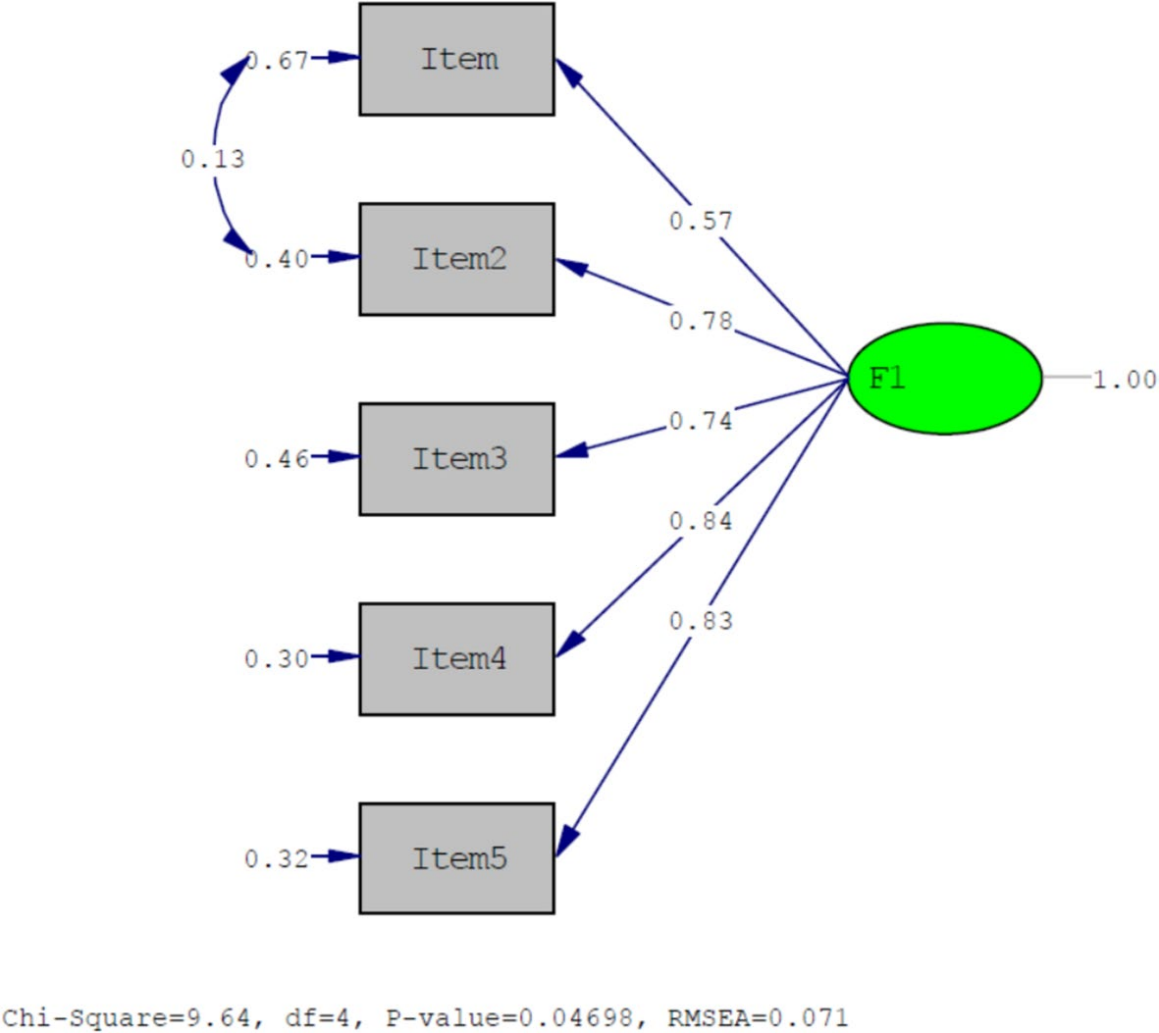
PATH diagram regarding the factor structure of the scale

According to the results of the analyses, CFA fit index values were found as follows: *X*^2^ = 9.64 df = 4 (*p* < 0.05), *X*^2^/df = 2.41, RMSEA = 0.071, CFI = 0.99, SRMR = 0.021, TLI = 0.98, RMR = 0.012, and AIC = 31.64 (Table 3). The PATH diagram created during the confirmatory factor analysis is shown in Fig. 1.

**Table 3:** Results of confirmatory factor analysis

Fit criteria	Found	Appropriate	Acceptable	Sonuç
*x*^2^/df (CMIN/DF)	2.41	< 2	< 5	Acceptable Fit
RMSEA	0.071	< 0.05	< 0.08	Acceptable Fit
CFI	0.99	> 0.95	> 0.90	Excellent Fit
SRMR	0.021	< 0.05	< 0.08	Excellent Fit
RMR	0.012	< 0.05	< 0.08	Excellent Fit
TLI	0.98	> 0.95	> 0.90	Excellent Fit
AIC	31.64			

*CFI* Comparative fit index, *RMSEA* Root mean square error of approximation, *RMR* Root mean square residual, *SRMR* Standardised root mean square residual, *TLI* Tucker Lewis index, *AIC* Akaike information citation

### Results Related to Reliability

The total Cronbach’s alpha value of the scale was 0.860. In addition, the Omega reliability value of the scale was found to be 0.866 (Table 2). When the item-total correlation coefficients of the scale were analysed, it was found that all item-total correlation coefficients were between 0.578 and 0.755 (Table 2).

During the research process, the correlation value observed as a result of the test–retest measurements applied to 30 people 15 days apart in order to evaluate the consistency of the scale over time was found to be *r* = 0.989 and statistically significant (*p* < 0.01). There was also no statistically significant difference between the test–retest measurement results (Table 4) (*p* > 0.05).

**Table 4:** Test–retest results and mean scores (*n* = 30)

Scale	Scale score means		Analysis results			
	First implementation *X* ± SD	Second implementation *X* ± SD	*r*	*p*	*t*	*p*
BV-SCSRFQ total	13.43 ± 4.10	13.30 ± 3.93	0.989	.000**	1.161	0.255

^**^Correlation is significant at the 0.01 level (2-tailed), *r* Pearson's correlation coefficient; *t*, *t* Paired sample *t* test, *BV-SCSRFQ* The brief version of the Santa Clara strength of religious faith questionnaire

## Discussion

In the study, the content validity index (CVI) value was found to be 0.98. According to the literature, a CVI analysis result of > 0.80 is considered sufficient for the content validity of the scale (Polit & Beck, 2008). The value of 0.98 indicates that the views of the specialists on the subject are largely consistent and the items measured are sufficient.

Before conducting factor analysis, suitability of the sample size and suitability of the data set for factor analysis were evaluated. For this purpose, KMO and Bartlett’s Sphericity Test were applied. Bartlett’s Test of Sphericity gave significant results. KMO value was found to be 0.850. In order to show the validity of the existing 5-item unidimensional structure, CFA analysis was made before EFA was applied. A factor loading of > 0.30 is a requirement for the scale to be acceptable (Finch, 2019; Seçer, 2020). According to the evaluation made by CFA analysis, it was found that the factor loading values ranged between 0.570 and 0.840. The condition that factor loading values should be > 0.30 was met and it was determined that the unidimensional structure was confirmed.

Fit indices of the scale were calculated as follows: *X*^2^ = 9.64, df = 4 (*p* < 0.05), *X*^2^/df = 2.41, RMSEA = 0.071, CFI = 0.99, SRMR = 0.021, TLI = 0.98, RMR = 0.012, AIC = 31.64. As a result of CFA, acceptable levels of fit indices were defined as CMIN/DF not exceeding 5, RMSEA and SRMR being lower than 0.08, and TLI being higher than 0.90 (Aksu et al., 2017; Bae, 2017; Erkorkmaz et al., 2013). Within this framework, it was determined that the fit indices of the scale were at adequate and excellent levels, and the uni-sub-dimensional structure including five items and its Turkish validity was confirmed.

Total Cronbach’s alpha value of the scale was 0.860, and Omega reliability value was 0.866. In the short version of the original scale, Cronbach’s alpha value was 0.95 (Plante et al., 2002).

Item-total correlation coefficients are calculated to evaluate the relationship of scale items with other items and total score, and it is accepted that this value should be > 0.30 (Finch, 2019; Seçer, 2020). When item-total correlation coefficients of the scale were analysed, it was found that all item-total correlation coefficients were between 0.578 and 0.755. The correlation coefficient is evaluated as follows: values of ≥ 0.80 indicate a high level of relationship, values between 0.60 and 0.80 indicate a strong relationship, values between 0.40 and 0.59 indicate a moderate relationship, values between 0.20 and 0.39 indicate a low relationship, and values of < 0.20 indicate a very weak relationship (Karasar, 2020). It can be seen that there is a strong relationship in line with the values found in the study. The correlation value observed as a result of the test–retest measurements was found to be r = 0.989, and no significant difference was observed between the test and retest results. In line with the results, it can be understood that the scale has a high level of reliability.

### Study Limitations

There are several limitations to this study that should be considered when interpreting the results. First, the sample size, while adequate for the statistical analyses conducted, may not be fully representative of the larger population, limiting the generalisability of the findings. Second, the use of online data collection may have introduced response bias because participation was limited to those with internet access and willingness to complete the survey. Finally, while effective for reaching the 18+ population, snowball sampling relies on participant networks and may unintentionally exclude some groups, further impacting the representativeness of the sample.

## Conclusion

As a result of the evaluation, it was confirmed that the Turkish version of the scale consisting of five items and a single sub-dimension was similar to the original version. Cronbach’s alpha values and fit indices were found to be in parallel with the original version. According to these results, it was found that the Turkish version was identical to the original scale, and thus, cultural equivalence was achieved. The results of the study prove the validity of the Turkish short version of the Santa Clara Strength of Religious Faith Questionnaire as a valid and appropriate measurement tool in the Turkish population.

## Data Availability

The data that support the fundings of this study are available on request from the corresponding author.
